# Abscessed Lumbar Hypodermatitis Revealing Pyonephrosis: A Diagnosis to Keep in Mind

**DOI:** 10.7759/cureus.104566

**Published:** 2026-03-02

**Authors:** Abdennasser Lakrabti, Ali Akjay, Jihad Anzaoui

**Affiliations:** 1 Urology, Moulay Ismail Military Hospital, Meknes, MAR; 2 Urology, University Sidi Mohamed Ben Abdellah, Moulay Ismail Military Hospital, Meknes, MAR; 3 Department of Urology, Faculté de Médecine, de Pharmacie et de Médecine Dentaire de Fès, Fez, MAR

**Keywords:** abdominal wall abscess, calculus, hypodermatitis, psoas abscess, pyonephrosis

## Abstract

Pyonephrosis resulting from an obstructing calculus commonly presents with symptoms such as loin pain, fever, and signs indicative of a urinary tract infection. In some cases, significant thinning of the renal parenchyma in pyonephrosis may lead to direct rupture into the retroperitoneum, and exceptionally rarely, into the lumbar abdominal wall, potentially mimicking an isolated abdominal wall abscess, which can be mistaken for a complication of hypodermatitis. Our case is a good illustration of a condition that is not well known among practitioners, particularly family physicians and dermatologists. This lack of awareness can explain the diagnostic delay, which may sometimes result in the patient's death.

## Introduction

Pyonephrosis is an infection of the upper renal-urinary system, which, over time, leads to suppurative destruction of the renal parenchyma [1]. Lithiasis is a common cause of this infection. Kidney stones are a common and urgent problem in urology, usually manifesting as acute colic. In some cases, urolithiasis remains asymptomatic for a long time and may manifest as complications such as lumbar wall abscess secondary to fistulization of pyonephrosis, which is a very rare complication reported in the literature.

## Case presentation

**Figure 1:**
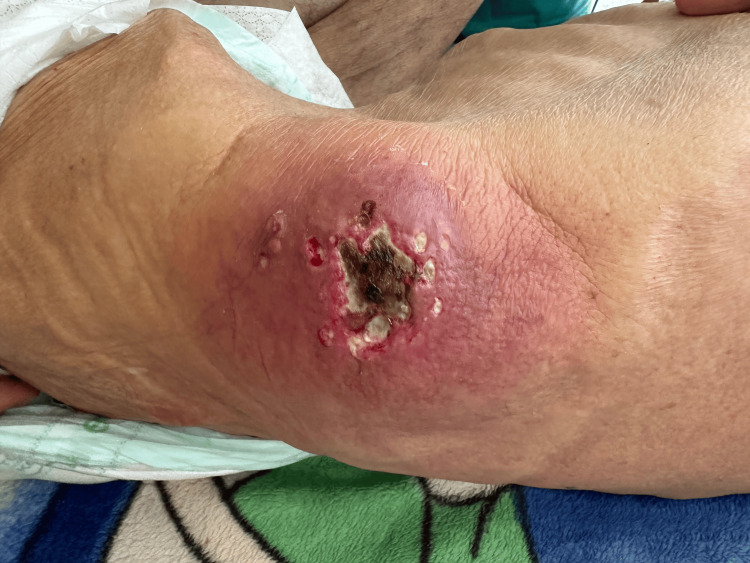
Left lumbar mass with overlying inflammatory skin

An 86-year-old man, with no notable pathological history, was admitted to the emergency department with swelling of the left lumbar abdominal wall (Figure 1).

The patient was treated for hypodermatitis of the lumbar region, but with no improvement under antibiotic therapy. In view of the increasing size of the lesion, he consulted the emergency department.

**Figure 2:**
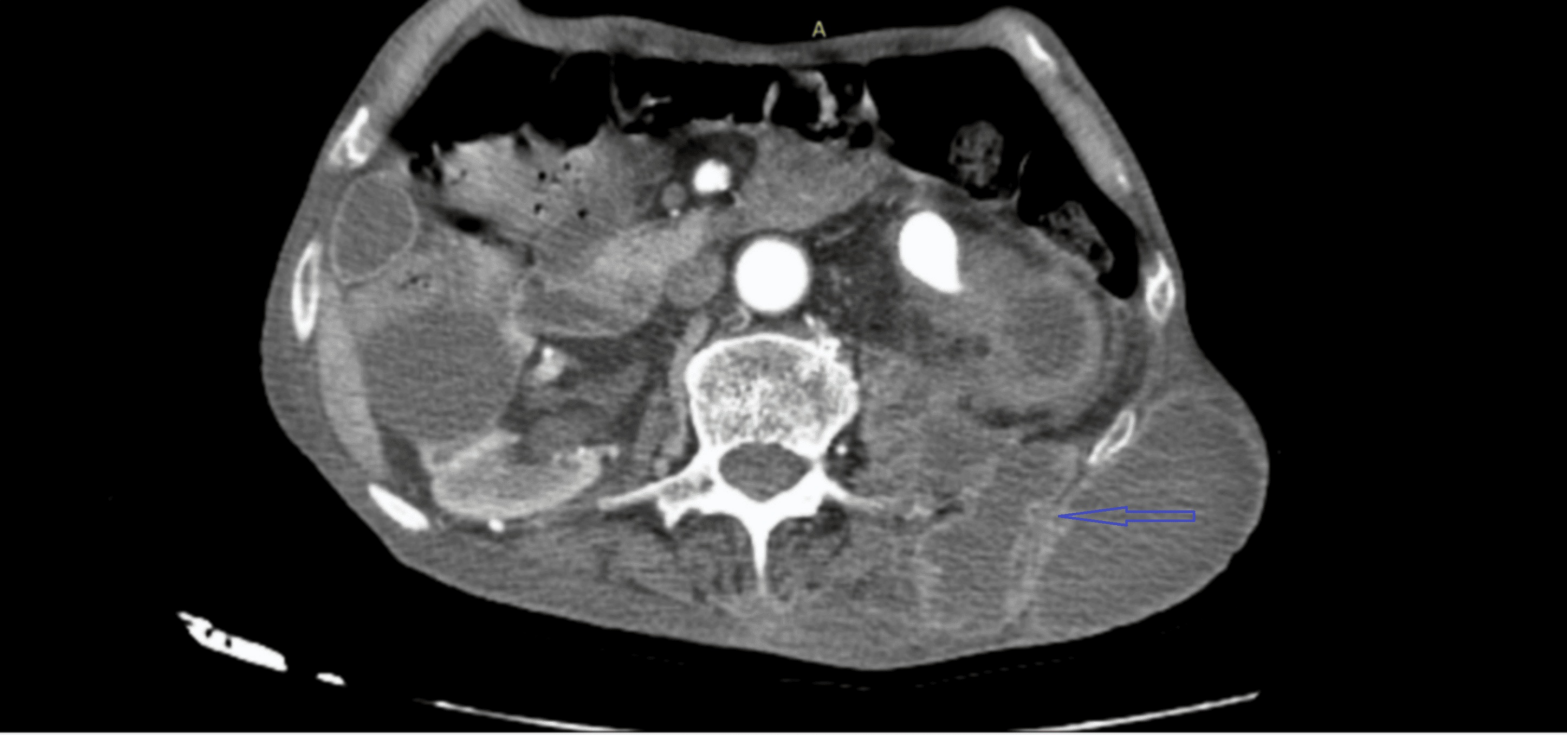
Axial image from the excretory phase of a contrast-enhanced abdominopelvic CT scan The image demonstrates left pyonephrosis caused by an obstructive stone in the renal pelvis, complicated by a retroperitoneal abscess with fistulization, leading to a subcutaneous lumbar collection in the same region indicated by the blue arrow.

**Figure 3:**
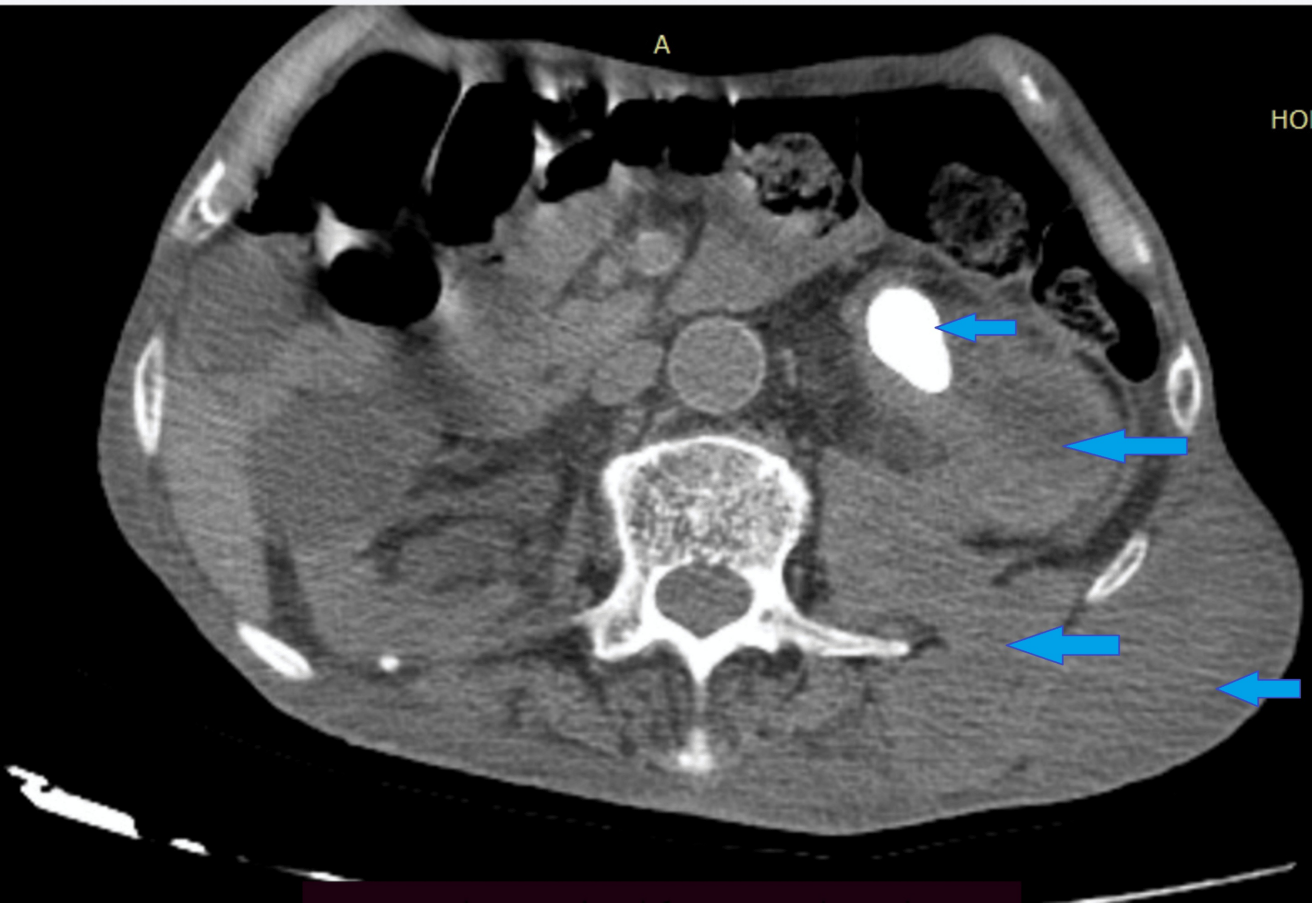
Axial image of an abdominopelvic CT scan The image reveals left pyonephrosis caused by an obstructive renal pelvic stone, complicated by a retroperitoneal abscess with fistulization, leading to a subcutaneous lumbar collection in the affected region, indicated by blue arrows.

Ultrasound and uroscanner revealed a renal pelvis calculus with pyonephrosis, a perineal abscess, and a psoas abscess extending to the left lumbar abdominal wall (Figures 2-3).

After percutaneous drainage, an Xpert® *Mycobacterium tuberculosis*/resistance to rifampin MTB/RIF test was carried out for tuberculosis, which proved negative. Biological parameters normalized and general condition improved, with recovery of autonomy after two months' treatment. A nephrectomy is planned to eliminate this undestroyed infection (Table 1).

**Table 1:** Laboratory parameters before and after drainage of the abscess

Biological parameters	Before drainage	After drainage
White blood cell, cells/mL	22000	8000
C-reactive protein (CRP), mg/l	260	30
Creatinine, mg/l	13	12
Hg, g/l	12	12

## Discussion

Nephrocutaneous fistula results from the spontaneous formation of an abnormal communication between the kidney and the skin. This fistula traverses the retroperitoneum and the structures of the abdominal wall following pathways of least resistance, such as Petit's triangle and the Grynfeld quadrilateral.

A retroperitoneal abscess, particularly a psoas abscess secondary to calyceal rupture in calculus-induced pyonephrosis, is exceedingly rare [2]. The detection of such a psoas abscess with computed tomography is nearly 100% [3].

In some instances, the abscess may extend into the muscles of the posterior abdominal wall, notably the quadratus lumborum, presenting as a lumbar abscess, as seen in our case. Management typically involves broad-spectrum antibiotics along with percutaneous or surgical drainage of pus via nephrostomy and ureteral stent placement [2]. Surgical removal of the affected kidney is often necessary, particularly when it is non-functioning or affects both the affected and normal-functioning contralateral kidney [4,5].

A thorough review of the existing French and English medical literatures identified fewer than seven reported cases of spontaneous rupture of pyonephrosis secondary to urolithiasis with psoas abscess formation [2,6,7]. In our case, an additional finding was the presence of a posterior abdominal wall abscess in the lumbar region, which has not been reported previously. However, there has been a reported case of lumbar panniculitis with a subcutaneous abscess secondary to pyonephrosis [6]. Additionally, literature describes rare occurrences of peritoneal rupture of pyonephrosis leading to peritonitis and splenic abscess [8,9].

Our case is a good illustration of a condition that is not well known among practitioners, particularly family physicians and dermatologists. This lack of awareness can explain the diagnostic delay, which may sometimes result in the patient's death. Our case is a good example of a pathology that should be considered by practitioners, whether general practitioners, dermatologists, or other specialists, to avoid any diagnostic delay.

## Conclusions

Given the increased variability in the symptoms of pyonephrosis, it becomes evident that early diagnosis is crucial. Spontaneous rupture of pyonephrosis is a rare occurrence. Abscesses of the lumbar abdominal wall are a rare complication of pyonephrosis. Accurate diagnosis and thorough examination to identify the source of the abscess are essential prior to intervention. Inclusion of a CT scan of the abdomen in the standard protocol for evaluation of all lumbar abscesses is necessary to exclude a renal origin of the abdominal abscess.
